# Unwavering Pathobiology of Volumetric Muscle Loss Injury

**DOI:** 10.1038/s41598-017-13306-2

**Published:** 2017-10-13

**Authors:** Sarah M. Greising, Jessica C. Rivera, Stephen M. Goldman, Alain Watts, Carlos A. Aguilar, Benjamin T. Corona

**Affiliations:** 1 Extremity Trauma and Regenerative Medicine, United States Army Institute of Surgical Research, Fort Sam Houston, TX USA; 2 Massachusetts Institute of Technology, Lincoln Laboratory, Lexington, MA USA; 3 Department of Biomedical Engineering, University of Michigan, Ann Arbor, MI USA; 4 Department of Physical Medicine and Rehabilitation, Uniformed Services University of Health Sciences, Bethesda, MD USA

## Abstract

Volumetric muscle loss (VML) resulting from extremity trauma presents chronic and persistent functional deficits which ultimately manifest disability. Acellular biological scaffolds, or decellularized extracellular matrices (ECMs), embody an ideal treatment platform due to their current clinical use for soft tissue repair, off-the-shelf availability, and zero autogenous donor tissue burden. ECMs have been reported to promote functional skeletal muscle tissue remodeling in small and large animal models of VML injury, and this conclusion was reached in a recent clinical trial that enrolled 13 patients. However, numerous other pre-clinical reports have not observed ECM-mediated skeletal muscle regeneration. The current study was designed to reconcile these discrepancies. The capacity of ECMs to orchestrate functional muscle tissue remodeling was interrogated in a porcine VML injury model using unbiased assessments of muscle tissue regeneration and functional recovery. Here, we show that VML injury incites an overwhelming inflammatory and fibrotic response that leads to expansive fibrous tissue deposition and chronic functional deficits, which ECM repair does not augment.

## Introduction

Biomaterial-based technologies that enhance host tissue regenerative responses hold significant promise for restoration of functional soft tissue. Traumatic partial ablation of skeletal muscle, or volumetric muscle loss (VML) injury is particularly in need of an effective regenerative therapy. VML injury presents a defect region in which all native elements required for canonical skeletal muscle regeneration (e.g., basal lamina and satellite cells) are removed; and, because adult mammalian skeletal muscle is not adept at *de novo* muscle fiber regeneration or hyperplasia, chronic loss of muscle tissue, persistent strength deficits, and disability manifest^1–4^. Encouraging results in animal models, in which implantation of dissociated native muscle elements necessary for canonical muscle fiber regeneration effectively promoted *de novo* muscle fiber regeneration and neuromuscular functional recovery^5–7^, highlight the potential of regenerative therapeutics. The challenge now is to delineate which of these native regenerative elements (or their surrogates) must be implanted to instruct and interact with the host *in vivo*.

Acellular biological scaffolds, or extracellular matrices (ECMs), are biological materials that have been widely evaluated for VML repair due to their off-the-shelf availability, and zero autogenous donor tissue burden. Various ECMs derived from different species and tissues using proprietary decellularization methods are in clinical use for several soft tissue indications, to include herniated abdominal muscle wall repair. While ECM devices are FDA approved for soft tissue reinforcement with general claims of host tissue deposition, which may potentially be fibrotic, the basic literature is comprised of numerous reports that have investigated if ECMs specifically promote functional muscle tissue regeneration (for review see^8^). Implanted ECMs are hypothesized to create a regeneration-permissive environment within the defect area that chemoattracts and modulates the myriad of cell types needed to orchestrate regeneration of functional skeletal muscle tissue. The existing literature reports mixed results on the capacity of ECMs to promote this form of skeletal muscle tissue specific constructive remodeling^9,10^. The potential of constructive remodeling, however is supported by an impressive report of a physiologically relevant magnitude of muscle tissue regeneration following an ECM implantation in a large animal model^11^.

A recent clinical trial^12^ involving 13 patients investigated commercially available decellularized small intestine submucosa (SIS) and urinary bladder matrix (UBM) derived ECM repair of VML injury to improve functional outcomes. Using muscle function as a metric of efficacy, in comparison to the injured limb pre-surgically 7 patients improved function, 3 had no change, and 3 had subsequent loss of function. ECM repair resulted in bulking of the muscle unit; however, further investigation using muscle biopsies does not conclusively present evidence of muscle fiber regeneration in the area of implantation beyond islands of muscle fibers (~10–50 muscle fibers). Moreover, these fibers are unlikely to significantly contribute to voluntary contractile force in human muscles, which are comprised of hundreds of thousands of muscle fibers^13,14^. Regardless, the stated conclusion drawn in this work is that biomaterial mediated constructive remodeling promotes *de novo* muscle tissue regeneration that improves limb function^15^.

The difficulty of powering clinical trials to determine efficacy and mechanism of action, matched with the discrepancies within the basic science literature in demonstrating ECM-mediated functional muscle tissue regeneration currently confounds clinical recommendation of ECMs for VML injury repair. Herein we performed a translational study in a porcine VML injury model to interrogate the capacity of commercially available ECMs to promote functional recovery of VML injured musculature and used unbiased analyses for functional recovery and regeneration of muscle tissue.

## Results

### Confirmation of Porcine VML Injury

**Figure 1:**
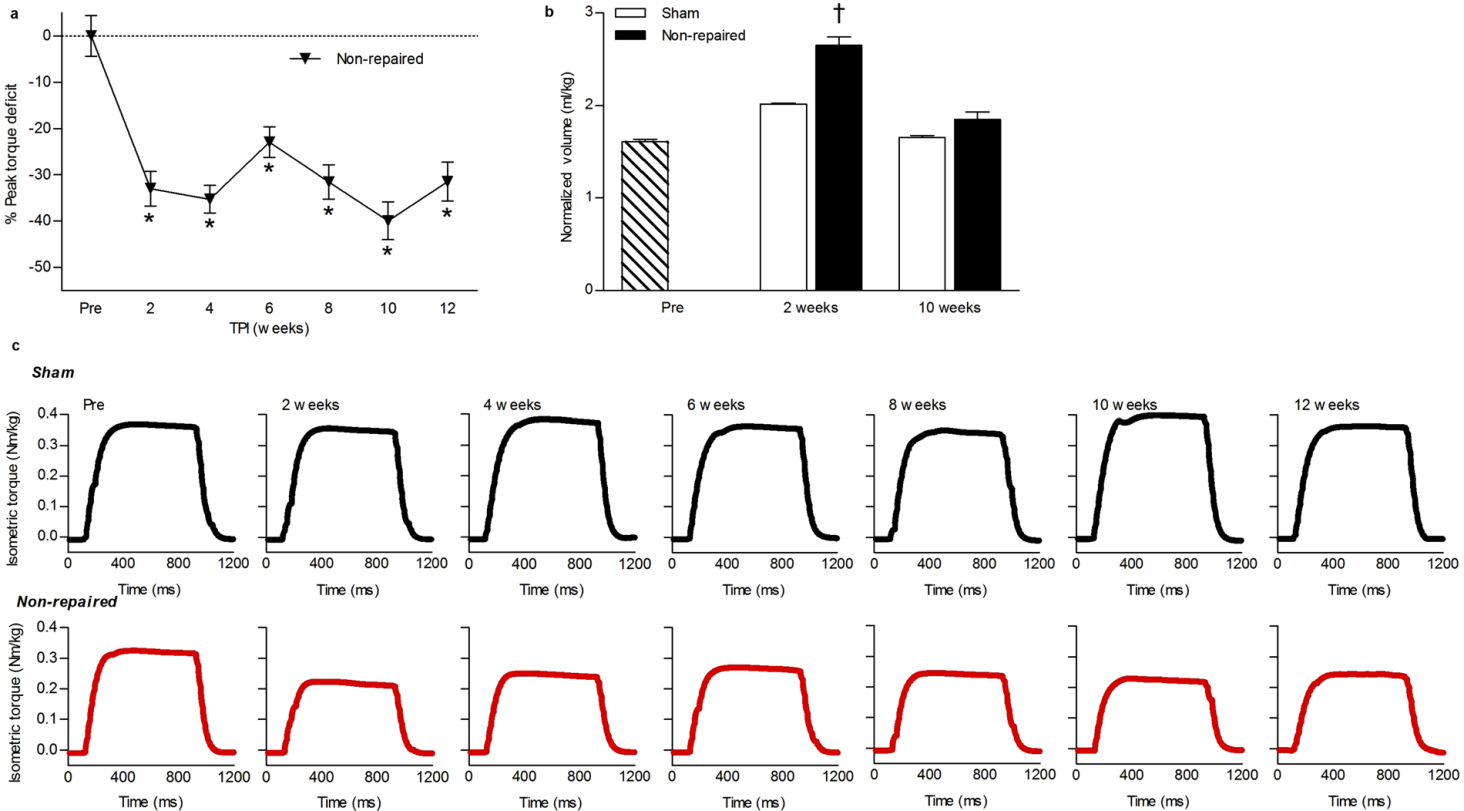
VML injury in the porcine PT muscle causes persistent functional deficits. (**a**) The peak strength deficit of the non-repaired (n = 12) compared to sham-operated control (n = 2) at each time point was determined. The significant torque deficits at each time point were compared to the pre-surgical time point (one way ANOVA p ≤ 0.001; *significantly different than combined pre). (**b**) The volume of the anterior compartment was normalized to body weight at each time point. At 2 weeks post-injury there is a significant increase (one way ANOVA p = 0.009; ^†^significantly different than sham at the same time point) in the volume of the compartment, likely indicative of swelling and inflammation in the non-repaired limb (n = 4) in comparison to the sham-operated (n = 2). (**c**) Representative peak isometric torque tracings for the sham (black) and non-repaired (red) groups are presented.

**Figure 2:**
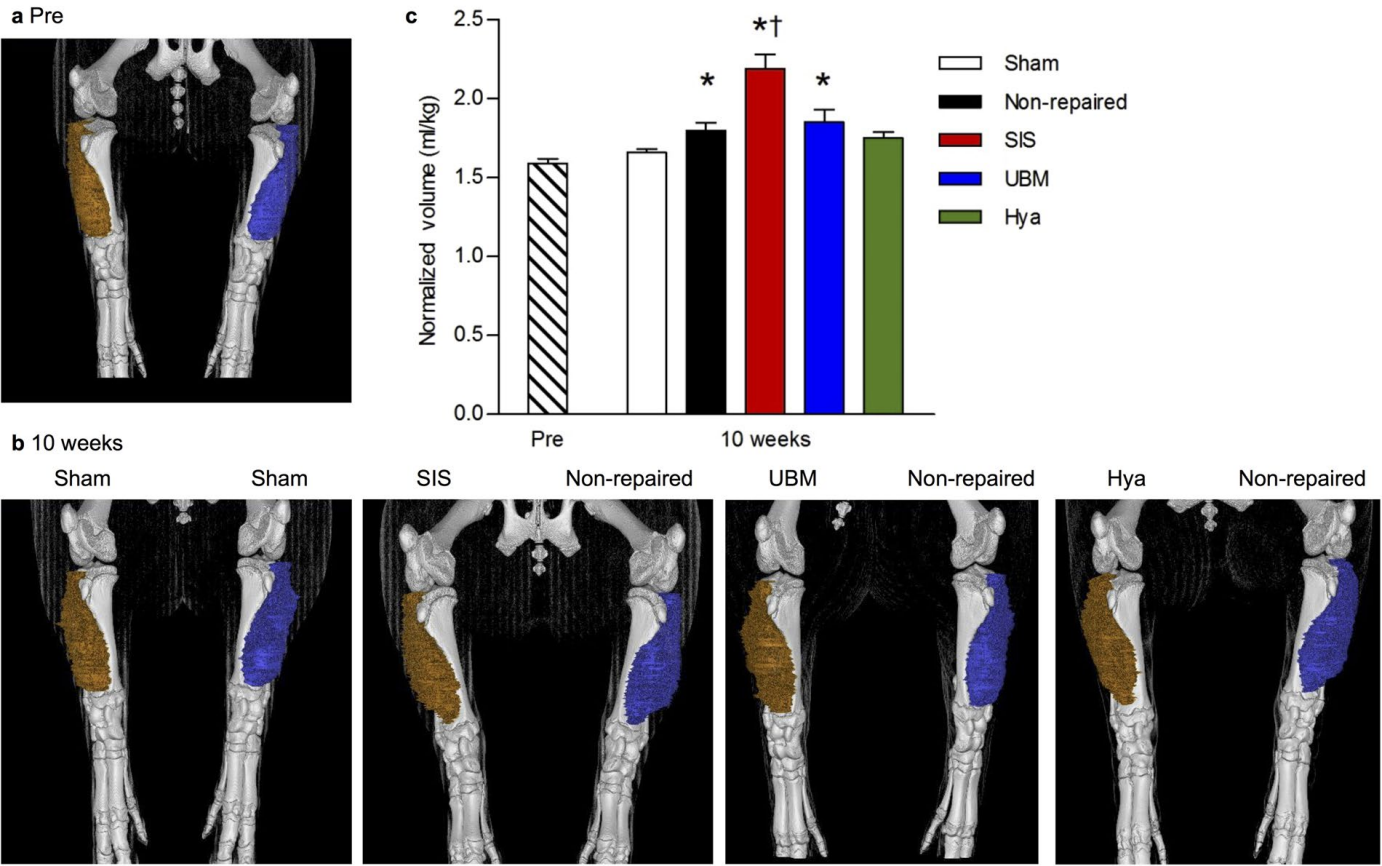
ECM repair induces tissue bulking within the anterior compartment. Standard imaging studies of the lower limb and specifically the anterior compartment were conducted with CT at 0.5 mm slice thickness; scans were acquired pre-surgically at 10 weeks post-injury. The 3D reconstruction of the limbs illustrates the anterior compartment volume for each experimental groups at the (**a**) pre-surgical and (**b**) 10 week scan. The volume of the anterior compartment normalized to body weight was not different between experimental groups and was averaged across all animals at the pre-surgical time point and is collapsed into one group (n = 12). (**c**) There was a significant increase in the volume normalized to body weight at 10 weeks, in particular the SIS-repaired group (n = 5) had the largest volume (sham n = 2; non-repaired n = 12; UBM-repaired n = 4; Hya-repaired n = 3, one way ANOVA, p < 0.001; significantly different than *combined pre; †non-repaired, UBM, and Hya).

**Figure 3:**
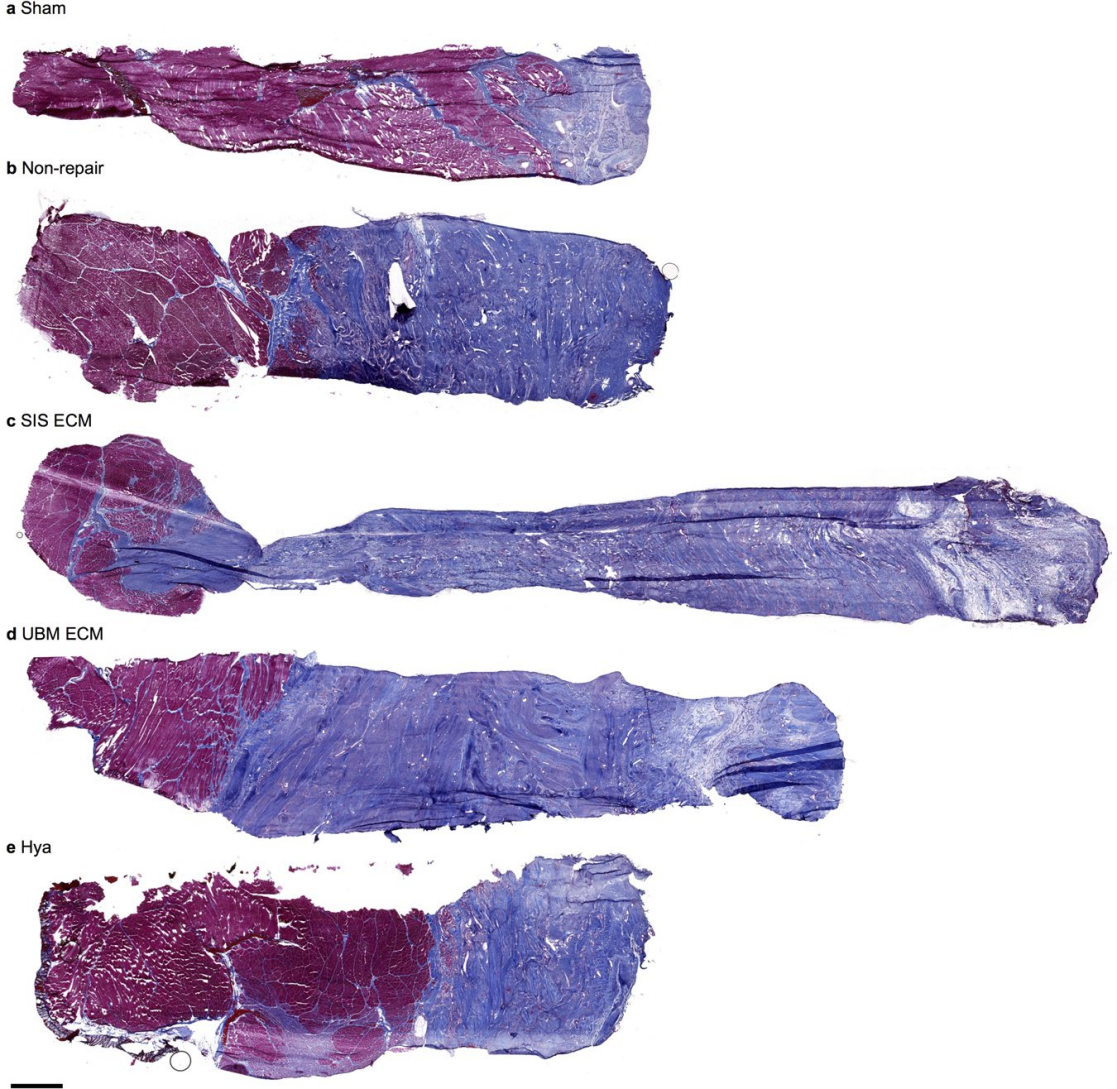
VML injury incites an overwhelming fibrotic response that is not alleviated by ECM repair. Histologic representation of a full thickness sample of porcine PT muscle through the VML defect area. Notably, there is a significant fibrotic response in the muscle, with more fibrosis repaired groups in comparison to the non-repaired. In all cases the increased fibrosis is infiltrating the native muscle tissue. In the repaired muscles there is no to merely non-physiologic islands of skeletal muscle fibers in the VML defect area. Additionally, islands of skeletal muscle fibers are also present in defect area of non-repaired muscles. Masson’s Trichrome stained (connective tissue is blue; nuclei are purple; skeletal muscle fibers are red) 12 weeks following (**a**) sham procedure or surgical repair of VML injury to the using a (**c**) SIS, (**e**) UMB, (**f**) Hya, or leaving the muscle (**b**) non-repaired. Scale bar is 2 mm; all images are at the same magnification.

VML injury is defined as partial ablation of skeletal muscle that cannot be endogenously regenerated and imparts a persistent functional deficit^16,17^. Herein, VML injury was surgically created bilaterally in the porcine peroneous tertius (PT) muscle, the primary dorsiflexor muscle, by excising 5.10 ± 0.03 g muscle tissue to create a defect of ~3 × 3 × 1 cm in the middle third of the musculature (Supplementary Fig. 1). The VML injury increased anterior compartment soft tissue volume compared to sham-operated values measured using CT by ~31% at 2 weeks post-injury (p = 0.009), likely due to acute inflammation, and later resulted in an ~8% increase at 10 weeks post-injury (Figs 1 and 2). The latter increase in soft tissue volume corresponded with histological evidence of extensive fibrotic tissue deposition with no clear indication of muscle fiber regeneration within the defect area (Fig. 3).

To quantify the physiological magnitude of VML injury, *in vivo* neural-evoked functional assessment of anterior compartment musculature was performed (Supplementary Fig. 2). Following VML injury without repair, a significant persistent 23–40% peak strength deficit was observed through 12 weeks post-injury compared to sham-operated control values (Fig. 1; p ≤ 0.001). Collectively, the gross fibrotic response and persistent functional deficit observed in this VML model reflects salient pathophysiological outcomes observed clinically^1,2^.

### Biomaterial Repair of VML injury

#### Hyaluronic Acid Hydrogel

As an initial step in determining the capacity of an ECM to promote constructive remodeling of skeletal muscle in a porcine VML model, we sought to establish a biomaterial negative control incapable of orchestrating ECM-mediated constructive remodeling. In previous rodent VML models, ECM implantation promoted functional improvements via putative improvements in fibrosis-mediated force transmission^18,19^, and therefore we also sought to establish this negative control for implant-mediated functional improvements that are not associated with skeletal muscle regeneration. Hyaluronic acid hydrogel (Hya) was selected because it lacks cell binding motifs and matricryptic peptides^20^ that are presumptively pivotal to scaffold mediated regeneration^9^. In a pilot study, Hya was implanted into a tibialis anterior (TA) muscle rat VML model and assessed for *in vivo* TA muscle strength as a function of stimulation frequency and muscle tissue regeneration 8 weeks post-injury. Non-repaired TA muscles presented a 46–61% strength deficit, which Hya did not improve at any stimulation frequency (Supplementary Fig. 3; non-repaired vs. Hya; p ≥ 0.165). Additionally, no evidence of muscle tissue regeneration within the defect region was observed, although Hya degradation was apparent from 2 to 8 weeks post-injury (9.8 ± 0.8 vs. 4.9 ± 1.6 mm^2^, respectively; p = 0.032). Lastly gene expression of inflammatory cytokines (IL-10, Tgf-ß1, and Tnfα) and myogenic transcription factors (myogenin and Pax7) key to skeletal muscle regeneration was analyzed in TA muscles 2 weeks post-injury to determine if Hya impacted the molecular phenotype of the healing response. Expression of all genes was similarly up-regulated in Hya and non-repaired muscles (p ≥ 0.421) when normalized to uninjured contralateral muscles. Collectively, these results supported Hya as a suitable negative control for further testing alongside selected ECMs in the porcine PT VML model.

#### Acellular Biological Repair of VML Injury

The remaining studies were conducted to determine if ECM repair (SIS or UBM) of VML injury improves neuromuscular strength and muscle tissue regeneration compared to injuries left non-repaired or repaired with Hya (biomaterial negative control). PT muscle VML injuries were repaired immediately by an orthopaedic surgeon and observed out to 12 weeks post-injury.

#### Gross Observations and Complications

Swelling was obvious within the initial weeks post-surgery in the non-repaired and UBM-repaired limbs compared to sham-operated limbs. Negligible swelling on par with sham-operated limbs was observed in the Hya-repaired limbs. SIS-repaired muscles presented greater swelling than all other conditions. 12 weeks post-VML injury, the majority of limbs displayed ambiguous muscle unit borders between the PT muscle and the lateral or medial synergist muscles in the anterior compartment. There was apparent tethering between the PT muscle and synergists muscle, fascia, and the skin. In all cases, the fibrosis was greatest on the lateral aspect of the limb. The fibrosis and the tethering throughout the lower limb was most pronounced in the SIS-repaired limb, in which often there were nodules or hardened aspects of the fascia outside of the muscle. During muscle harvesting, the fibrous tissue bled extensively. In contrast to the ECM-repaired limbs, discrete muscle boundaries within the anterior compartment were observed and tissue tethering was present but markedly reduced following repair with Hya.

Notably, following the initial surgery and repair, unexpected and significant complications developed after SIS ECM-repair (Supplementary Fig. 4). At the 2–3 day post-surgical bandage change, incisions were healing but the SIS-repaired limb had notably greater swelling and erythema than all other groups, particularly the contralateral non-repaired limb. All SIS-repaired limbs displayed prolonged inflammation and swelling for the duration of the study and 80% of the limbs displayed wound complications for 8–28 days. Necessary treatment was provided to all pigs with complications, ranging from wound irrigations, debridement, additional medications, and partial removal of exuding biomaterial components. No major adverse events were noted in the sham, non-, UBM-, or Hya-repaired limbs, although one non-repaired limb had a minimal wound dehiscence, with no additionally noted swelling. It is unclear why the SIS device presented complications; given that no complications were noted in the contralateral non-repaired limb, the order of limb operation was randomized, and one of the pigs in this group did not present complications, it is not likely that compromised surgical sterility or device preparation was involved. It is possible that the surgical strategy for SIS repair, which was derived from a previous case study^2^ and involved implantation of three overlying sheets, introduced too great of a biological burden and caused wound dehiscence. Importantly, no adverse outcomes were reported using SIS or any other ECM device in a recently completed clinical trial of VML repair^15,21,22^.

#### Anterior Compartment Volume and Tissue Formation

To assess the magnitude of tissue bulking promoted by ECM implantation, the volume of the anterior compartment of the lower limb was measured using standard CT imaging and normalized to body weight (Fig. 2). Across all limbs, pre-surgically the average volume of the anterior compartment was 1.6 ± 0.1 ml/kg body weight and was not different than sham-operated limbs assessed at 10 weeks post-injury. Tissue bulking of the anterior compartment was most prominent in the SIS-repaired limb, which displayed ~22% greater volume than the non-repaired limbs (Fig. 2; p < 0.001). In contrast the UBM- and non-repaired limbs had similar volumes, while the Hya-repaired and the sham-operated were similar and lesser than all groups at 10 weeks post-injury.

Histological examination of the PT muscle indicated similar responses when the VML injury was left non-repaired or was repaired (Fig. 3). Qualitative analysis at multiple levels of the PT muscle consistently presented widespread fibrotic tissue deposition. The transitions from the remaining underlying muscle to bands of collagen were either abrupt with juxtaposed densely arranged mature collagen or more gradual with interweaving collagen interspersed with degenerating muscle fibers. However, there was no uniformity among repair groups. These observations were confirmed by independent pathologic analysis, which primarily indicated that the fibrotic tissue overlying the injured musculature was comprised of a heterogeneous composition of immature, loosely arranged, and interweaving mature collagen fibers. The overlying fibrosis also contained noted perivascular inflammation (both lymphocytes and histiocytes) and prior hemorrhage, with no apparent differences across non- or repaired limbs.

**Figure 4:**
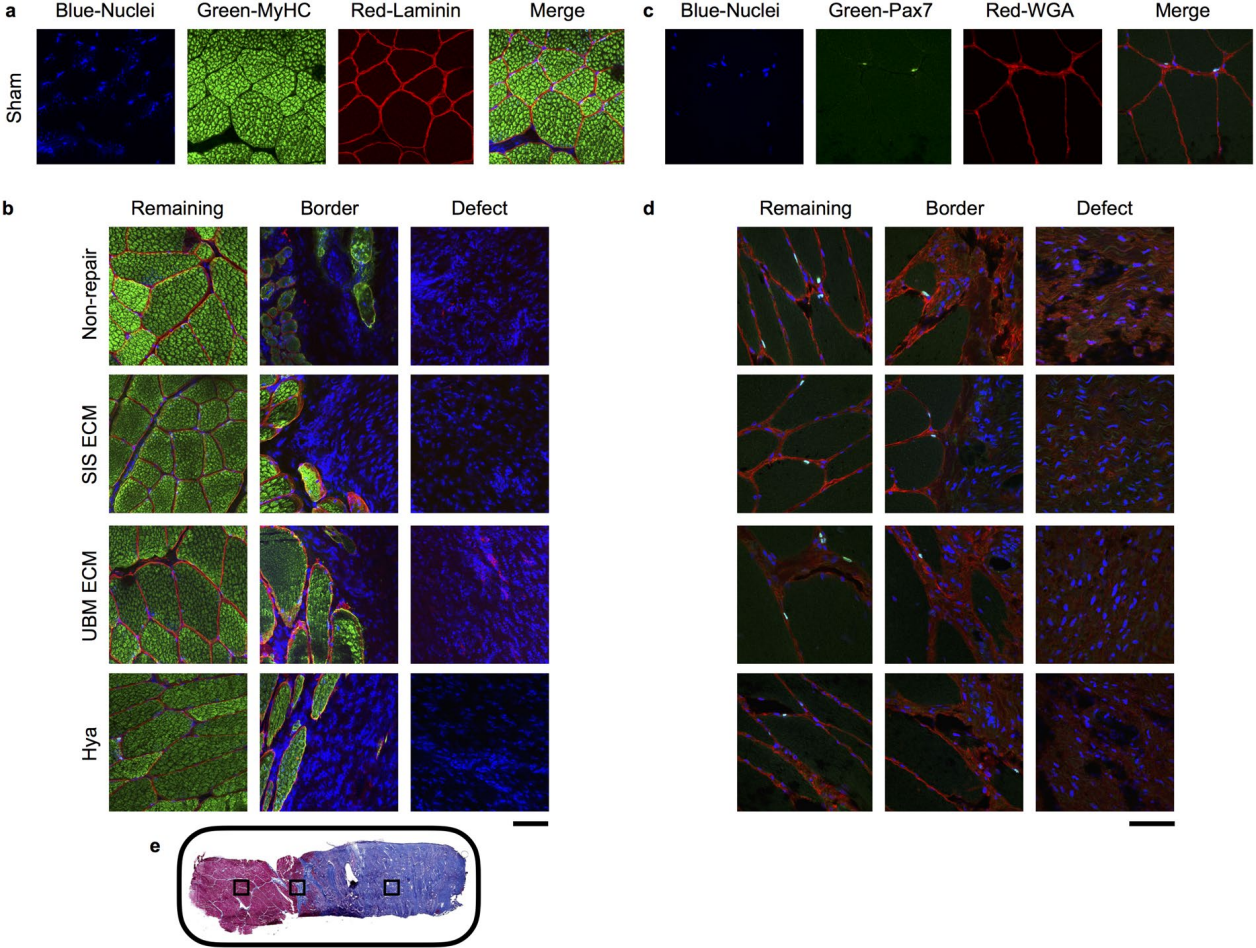
ECM repair presents no evidence of sustained muscle regeneration within the VML defect. Representative histologic images labeled with laminin (fiber sarcolemma), myosin heavy chain (MyHC, muscle fiber) and DAPI (nuclei), or Pax7 (satellite cell), wheat germ agglutinin (WGA), and DAPI. (**a** and **c**) For each of the sham muscles individual staining and the merged image are presented, to illustrate individual staining and the combined imaged. (**d** and **e**) Examples for each group, specifically the non-, SIS-, UMB-, and Hya-reparied are shown. Collectively, the muscle remaining appears healthy in all groups and there is muscle fibers and satellite cells at the border of the muscle and the defect. However, no evidence of muscle fibers for satellite cells was present in the defect area. (**e**) A general schematic of image locations using the non-repaired section displayed in Fig. 3; in each sample, representative sections were imaged in the remaining muscle, the boarder between the muscle and the fibrosis, and the fibrotic defect area as demonstrated. Scale bar is 150 µm; all images are at the same magnification.

In regards to skeletal muscle fiber presentation, islands or pockets of less than 100 myosin heavy chain (MyHC) positive muscle fibers were observed in close proximity to the remaining musculature in each group; however, muscle fibers were not present throughout the defect (Fig. 4). Moreover, laminin, a primary component of the basal lamina required for skeletal muscle regeneration, was absent within the defect area of all groups with the exception of UBM-repaired muscles. In UBM-repaired muscles, wavy laminin structures were observed in the defect area that did not resemble the typical presentation in skeletal muscle, as observed in the remaining muscle mass of VML injured muscles. Given that the UBM device is comprised of basement membrane components, it is likely that the laminin protein is a remnant of the implanted ECM.

#### Defect Cellular Infiltration

**Figure 5:**
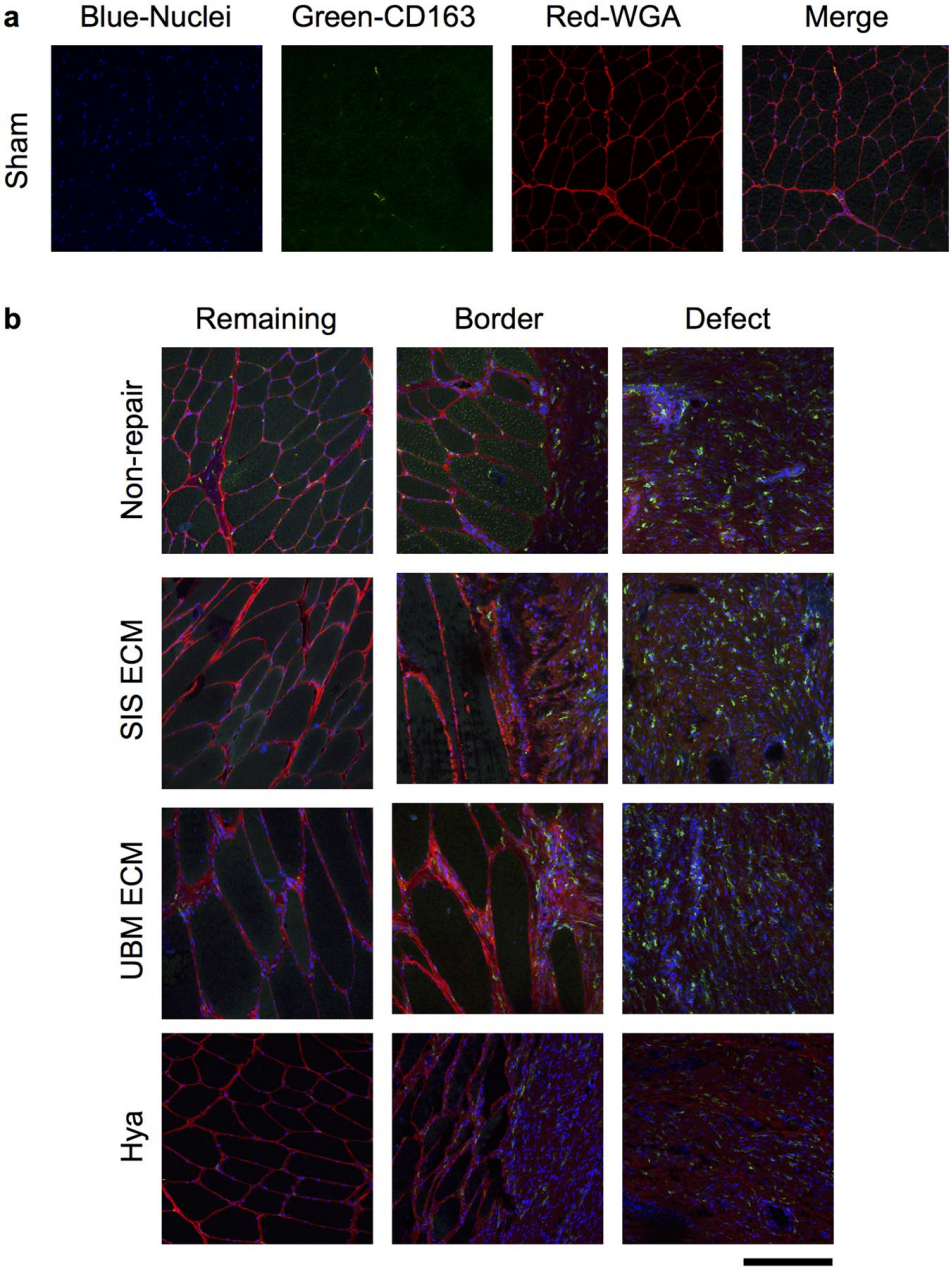
Non-repaired and repaired VML injured muscles present sustained putative macrophage infiltration. Representative histologic images labeled with CD163 (pan macrophage marker) and wheat germ agglutinin (WGA) and DAPI (nuclei). (**a**) For each of the sham muscle sections individual and merged images are presented. (**b**) Examples for each group, specifically the non-, SIS-, UMB-, and Hya-reparied are shown. Scale bar is 150 µm; all images are at the same magnification and follow the same location pattern displayed specifically in Fig. 4e.

Theoretically, an implanted ECM facilitates recruitment of myriad cell types in a spatiotemporal manner that is permissive to the well-characterized events of adult skeletal muscle regeneration^8,9^. We therefore performed immunohistological analysis of full-thickness PT muscle sections to gain insight into which cell types successfully migrated and survived in the defect area 12 weeks post-injury (Figs 4 and 5). There was no presentation of Pax7 positive nuclei (i.e., satellite cells) in the defect region of no repair, UBM, and Hya groups. Similarly, the defect region of SIS repaired muscles was largely devoid of Pax7 positive nuclei; however, in two SIS-repaired samples a small cluster of Pax7 positive cells were observed in close proximity to the remaining musculature (Supplemental Fig. 5). No evidence of muscle fiber regeneration was observed in association with these cell clusters. As expected, Pax7 positive satellite cells were observed in association with the basal lamina surrounding muscle fibers in the remaining muscle mass of all groups. Cells labeled with a porcine pan macrophage marker (CD163) were abundant in the fibrotic tissue deposited in the defect region in those limbs treated with either SIS or UBM or left non-repaired, and qualitatively lesser so in Hya-repaired limbs. Collectively, the histological phenotype of the PT muscle defect when repaired with SIS, UBM, or Hya or left non-repaired indicates an overwhelming deposition of a fibrous tissue devoid of muscle fibers, marked by prolonged macrophage infiltration and virtually no satellite cell presence.

#### Neuromuscular Strength

**Figure 6:**
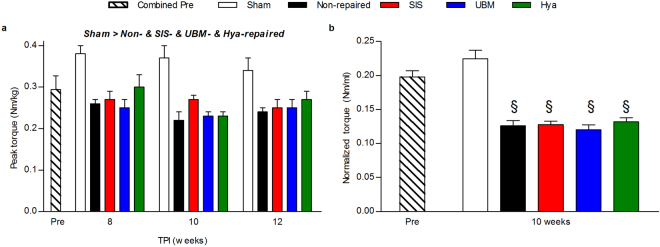
ECM repair failed to induce prolonged improvements of muscle strength. Muscle function determined by peroneal nerve stimulation *in vivo* was compared at 8, 10, and 12 weeks post-injury across all surgical groups. The torque of the anterior muscle compartment (normalized to body mass) was analyzed prior to surgery and was not different between experimental groups (p = 0.575); as such it was averaged across all animals at the pre surgical time point (n = 28). (**a**) At 8, 10, and 12 weeks post-injury muscle function across joint angels is presented. Across time there were significant main effects were determined in peak torque (sham n = 2; non-repaired n = 12; SIS-repaired n = 5; UBM-repaired n = 4; Hya-repaired n = 3; p < 0.001) specifically, the sham group was greater than all other experimental groups. There was not a main effect of time (p = 0.275) or interaction (p = 0.893). (**b**) The peak torque of the anterior compartment was normalized to an indicator of muscle size, specifically volume of the anterior compartment at 10 weeks post-injury. There was a significant deficit in all experimental group compared to the sham operated (pre n = 12; sham n = 2; non-repaired n = 12; SIS-repaired n = 5; UBM-repaired n = 4; Hya-repaired n = 3; p < 0.001; §significantly different than combined pre and sham). It is important to note that repair with the either the SIS or UBM ECM resulted in functional deficits that were equivalent to not repairing the VML.

The ultimate goal of any therapy for VML injury is to improve skeletal muscle strength. In rodent models, ECMs have improved skeletal muscle strength principally through improvement of force transmission as opposed to force generation^18,19^. Therefore we continued to interrogate the impact of ECM implantation on anterior crural muscle functional capacity by measuring maximal isometric tetanic torque *in vivo* at 8, 10, and 12 weeks post-injury. No differences in skeletal muscle function among experimental groups were observed before surgery. Post-injury, all VML groups regardless of repair had significant functional deficits compared to sham-operated limbs (Fig. 6; p < 0.001); a ~30% deficit (average across 8–12 weeks post-injury) in peak torque was observed regardless of repair. There was no significant difference over time (p = 0.275) and a significant interaction between group and time was not observed (p = 0.893). Peak muscle torque was also normalized to anterior compartment volume measured with CT at 10 weeks post-injury, to assess the quality of the functional capacity of the affected musculature. Regardless of repair following VML there was an ~43% deficit in torque normalized to anterior compartment volume compared to sham-operated controls (Fig. 2; p < 0.001). That is to say that repair of VML injury with SIS, UBM, or Hya resulted in no improvements of maximal neural-evoked functional capacity compared to leaving the injured muscle untreated.

#### Transcriptional Tissue Analysis

**Figure 7:**
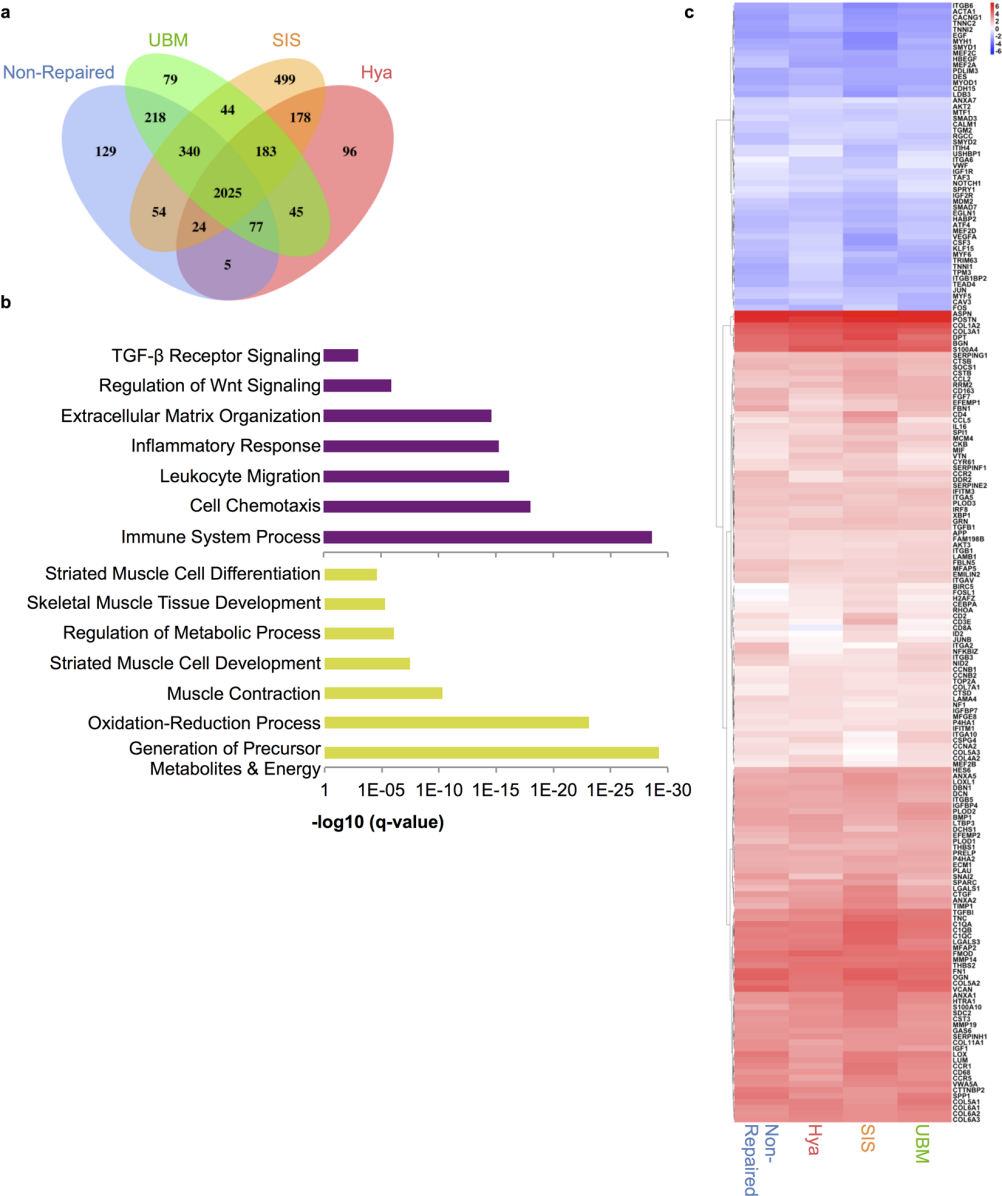
VML injured muscle presents prolonged degenerative transcriptional programs that are not ameliorated by ECM repair. (**a**) Venn diagram illustrating unique and overlapping genes obtained from different conditions. (**b**) Bar graphs of false-discovery rates of up- (purple) and downregulated (gold) over-represented pathways derived from differentially expressed genes. (**c**) Hierarchically clustered heatmap of relative expression values for the most statistically significant genes. The values are plotted as the average fold change in expression values relative to shams for the different treatment groups (sham n = 2; non-repaired n = 2; SIS-repaired n = 3; UBM-repaired n = 3; Hya-repaired n = 2).

Lastly, to quantitatively discriminate ECM-mediated modulation of the prolonged pathobiology of VML injury, expression profiling by RNA sequencing (RNA-seq; Fig. 7) from PT muscle tissue harvested 12 weeks post-injury was performed. Biological replicates from sham-operated, SIS-, UBM-, Hya-, and non-repaired limbs demonstrated excellent reproducibility (R^2^ > 0.95) and 11,506 total mRNAs were detected. In total, 3,996 genes were differentially expressed when compared to sham-operated, and the SIS-repaired displayed the highest number of differentially expressed genes compared to other repaired and non-repaired muscle (Fig. 7). Principal component analysis of the datasets from the treatment groups indicated significant separation of the sham from those injured by VML regardless of treatment (Supplemental Fig. 6). Additionally, strong correlations amongst tissues treated with a VML injury were observed, regardless of surgical repair with an ECM scaffold. These results suggest the gene expression programs induced from a VML injury are not appreciably augmented at the tissue level when surgically repaired using a biomaterials approach. Pathway analysis of the common and unique differentially expressed genes for VML-injured tissues was performed and gene set enrichments associated with immune-response, inflammatory signaling and ECM remodeling were observed, respectively (Fig. 7). Previously, sustained inflammation during muscle regeneration has been shown to drive degeneration and fibrosis^23,24^, which in turn prevents myogenic repair^25^. In line with this, upregulation of TGF-β and Wnt signaling pathways^26^ and increases in fibrogenic transcripts were viewed for each of the VML-treated tissues as well as relative downregulation of multiple myogenic development genes when compared to sham-operated controls (Fig. 7). The differentially expressed transcripts that were unique to each VML-injured tissue treatment did not yield any statistically significant pathways. Amalgamation of these findings suggests VML causes a universal pattern of expression driven by inflammatory and fibrotic gene sets and that repair by different types of ECM did not impact these signatures.

## Discussion

We unequivocally demonstrate that ECM implantation does not orchestrate skeletal muscle regeneration of a magnitude that imparts physiological improvement. Instead of functional muscle tissue regeneration, a remarkable deposition of fibrotic tissue was observed within the defect region three months post-injury. The fibrotic tissue was abundantly populated by macrophages, ostensibly fibroblasts, but was devoid of satellite or muscle fiber cells. This tissue phenotype is similar to that reported in a recent clinical trial^12,15^. Apart from small groupings of muscle fibers near the border of the remaining muscle mass that may have regenerated, no structural features of mature or regenerating skeletal muscle fibers were evident in the region of ECM implantation. ECM implantation did not ameliorate, and in some cases exacerbated, fibrotic tissue deposition compared to non-repaired VML injured muscles. Most importantly, ECM implantation did not improve the loss of *in vivo* nerve evoked muscle strength presented by non-repaired VML injured muscles out to three months post-injury. Transcriptome analysis indicated that ECM implantation neither augmented regenerative pathways nor repressed inflammatory or fibrotic pathways that defined the prolonged non-repaired response to VML injury, diminishing the likelihood of any delayed muscle regeneration after three months post-injury. Moreover, the regenerative, transcriptional, and functional outcomes were similar between muscles repaired with ECM and those repaired with Hya, which does not possess the properties theorized to support ECM mediated constructive remodeling.

ECMs are thought to promote skeletal muscle regeneration through a sequence of events that resemble, but may not completely mimic adult skeletal muscle regeneration^9^. While significant emphasis has been given to the macrophage and T lymphocyte responses to ECMs implanted in VML defects^27^, little attention has been given to identifying the putative myogenic precursor cell that migrates to and takes residence within the implanted ECM. Since, endogenous skeletal muscle fiber regeneration is absolutely dependent on satellite cell activity^28,29^, which differentiate and replenish themselves in response to injury, Pax7 positive cells should reside in areas of lasting skeletal muscle regeneration. In contrast, Pax7 positive cells were absent throughout the defect area three months after ECM implantation. The current study does not delineate if the absence of Pax7 positive cells in the defect region three months post-injury is due to an inadequate initial migration, reduction in proliferation, or a later selected death of satellite cells within the defect area. Rodent VML studies have demonstrated virtually no Pax7 positive cell presence within the defect two weeks after implantation of various biomaterials, pointing to material-induced fibrous encapsulation and/or inadequate chemoattraction as primary deficiencies in host satellite migration^1,19,30^.

Previous preclinical observations in rodent models of VML reported ECM-mediated improvements in nerve-evoked muscle strength. For instance, rodent studies using the same commercial UBM^19^, a custom-made UBM^31^, a syngeneic muscle derived ECM^18,32^, or an autologous devitalized muscle scaffold^33^ have observed moderate, though sometimes transient functional improvements. Notably, the functional improvements observed previously were not explained by *de novo* muscle fiber regeneration. For example, Aurora *et al*. counted the number of fibers within the defect area and reported regeneration of less than 2% of the muscle fibers lost (i.e., up to 50 muscle fibers regenerated^19^ of ~3,500 lost^34^). Thus, an alternative mechanism of functional recovery was proposed in rodent VML injuries, wherein ECM-related fibrous tissue deposition improved the transmission of forces produced by the remaining muscle mass (i.e., ‘functional fibrosis’)^18,19^. We therefore anticipated that ECM-repaired muscles would impart a modest functional improvement and that this mode of functional improvement could therefore explain the modest gains in isometric or isokinetic strength observed clinically^2,12^. However, the current findings do not support any mechanism of strength improvement and suggests that the putative mechanism of ECM improved force transmission is not conserved in larger VML defects. The drastic difference in fibrotic tissue deposition between porcine and rat VML models may account for this discrepancy (*see* Fig. 3 *&* Supplementary Fig. 3). In support, delivery of antifibrotic agent (Losartan) unexpectedly reduced TA muscle strength eight weeks after VML injury in the rat TA muscle^35^. The current findings indicate a need to revisit the therapeutic potential of antifibrotic therapies for VML injury that more aptly present clinical manifestations of fibrosis, such as the porcine model. In regards to the functional recovery discrepancy between these large animal findings and those in the clinic, it is important to note that the animal neuromuscular assessments involve maximal nerve stimulation (all motor units are activated) and therefore by-pass voluntary muscle recruitment strategies, while human strength assessments are voluntary and dependent on recruitment characteristics. It is therefore possible that the modest strength gains in the clinic may be due to neuromuscular recruitment adaptations that occur in the absence of *de novo* muscle tissue regeneration or even skeletal muscle hypertrophy.

In contrast to previous muscle regeneration studies^36,37^, where increases in expression of inflammatory and ECM remodeling genes temporally subsided after injury^38^, VML induces overexpression of these transcripts up to three months after injury. Detection of these transcripts was consistent with histological observations of macrophage infiltration into the VML defect site. Macrophages have been shown to secrete pro-fibrogenic factors such as TGF-β1 and upregulate components of the Complement system to perform phagocytosis after muscle injury. Both of these elements were significantly upregulated and enhance Wnt signaling, which has also been shown to attenuate satellite cell proliferation and enhance fibroblast production of collagen^39^. Thus, sustained activation of these gene expression programs from a VML injury may contribute to a poor environment for satellite cells and hinder their repair of the defect site, regardless of increased progenitor recruitment from an ECM. Since none of the ECMs directly impacted these expression programs, the underlying pathogenesis of VML was effectively unaltered and produced nearly indistinguishable transcriptomes at the tissue level. Collectively, the gene expression programs observed support a model whereby incessant inflammation from infiltrating macrophages results in over-activation of Complement, which in turn initiates Wnt signaling in mesenchymal progenitors and fibrogenesis^26,40^. This model of chronic inflammation resulting in defective satellite cell responses, enhanced proliferation of matrix-producing cells and increased ECM deposition has also been observed in muscular dystrophy^41^ and is uniquely consistent with other models of muscle tissue degeneration and fibrosis^25^.

### Outlook

The current observations are in line with the low magnitude of muscle fiber regeneration presented after VML repair in a host of small and large animal models^15,19,42,43^, but remain at odds with the robust regeneration presented by Turner *et al*.^11^. While large-scale tissue regeneration is challenged by the absolute distances required for revascularization and reinnervation, the fairly consistent observation of sub-physiological muscle regeneration following ECM repair for VML in both small and large animal models challenges the broader efficacy of ECM mediated muscle tissue regeneration^9^. The current study unequivocally indicates that ECM repair provides no functional benefit and suggests a unique pathology of chronic inflammation and persistent connective tissue deposition which may inhibit satellite cell based repair. The sub-physiological level of muscle tissue regeneration observed herein with any of the biomaterials implanted supports the continued development of biomaterial-based therapies that utilize biological adjuncts^44–46^ to potentially achieve a functionally meaningful magnitude of muscle tissue regeneration. However, the unwavering pathobiology of sterile VML injury observed herein regardless of immediate biomaterial repair draws significant concerns regarding the prolonged health of the remaining musculature and suggests that therapeutic intervention may be better served at attenuating pathology within the remaining musculature, as opposed to focusing solely on *de novo* muscle tissue regeneration. Collectively, this study argues that the use of ECMs for functional muscle repair following VML injury clinically is at best premature.

## Methods

### Animals

Female Yorkshire Cross pigs (n = 13) were purchased form Midwest Research Swine (Gibbon, MN). In pilot studies, male Lewis rats (n = 20) were purchased from Harlan Laboratories (Indianapolis, IN). All protocols and animal care guidelines were approved by the United States Army Institute of Surgical Research Institutional Animal Care and Use Committee (A-14-018 & A-14-017), in compliance with National Institute of Health Guidelines and the United States Department of Agriculture. All studies were conducted in compliance with the Animal Welfare Act, the Implementing Animal Welfare Regulations and in accordance with the principles of the Guide for the Care and Use of Laboratory Animals.

### Study Design

Studies were conducted to test currently clinically utilized ECMs and a biomaterial negative control. At the time of surgery bilateral VML injuries (or sham procedures) were performed and then each limb was randomized to no repair or implantation with a commercially available ECM or Hya (Supplementary Fig. 1). Pigs underwent initial functional testing prior to surgery and repeated testing up to every two weeks following surgery. Before and at 2 and/or 10 weeks after surgery, CT imaging of the lower hindlimb was performed. Terminally muscle samples were used for histologic and RNA analysis of the tissue. Pigs in all surgical groups (sham or VML) gained a significant amount of weight over the 12 week study; the body weight at the time of surgery and 12 weeks post-surgery was 44.1 ± 1.0 and 75.9 ± 1.5 kg, respectively. Weight gain was consistent and approximately 2.5 kg per week. At the end of the study, 12 weeks post-surgery, the pigs were 6.3 ± 0.1 months of age, considered to be young-adult.

In the pilot study to understand the biomaterial negative control, Hya, rats were randomized to unilateral VML injuries to the TA muscle that were left non-repaired or Hya-repair. At the time of surgery rats were 4.1 ± 0.1 months of age and weighed 367.4 ± 4.9 g, considered to be adults.

### Surgical Procedure

#### Volumetric Muscle Loss (VML)

While under isoflurane anesthesia and appropriate aseptic conditions, a ~3 × 3 × 1 cm area of muscle (5.10 ± 0.03 g) was surgically removed using sharp dissection from the middle third of the PT muscle (Supplementary Fig. 1). The fascia and skin were closed in layers with absorbable suture and compressive bandages were wrapped around each limb. Post-surgical analgesic administration of Buprenorphine SR (0.01 mg/kg, s.c.), Rimadyl (4.4 mg/kg, s.c.), and Excede (5.0 mg/kg, s.c.) was conducted through one week post-surgically. Sham operated limbs were surgically exposed and isolated, but no tissue was removed (Supplementary Fig. 1). In all cases the incision was closed in the same fashion. Following surgery, all animals recovered promptly and within 24 hours displayed normal eating habits, mobility and cage activity with only limited deficits in movement. No unexpected deaths occurred in any of the experimental animals.

In the rodent pilot study, while under isoflurane anesthesia a unilateral full-thickness VML injury was created using a 6 mm punch biopsy to the middle third of the TA muscle. On average, the tissue removed from the TA weighed 90.5 ± 3.2 mg. All rats received a one-time pre-surgical administration of buprenorphine-SR (1.2 mg/kg; s.c.) for pain management. Rats were randomized to repair with Hya or left non-repaired. Data was compared to the contralateral non-injured limb at 2 or 8 weeks post-surgery.

#### Biomaterial Implantation

The first biologic surgical mesh (Biodesign® 4 Layer Tissue Graft, G12579 C-SLH-45-7 × 20; Cook Medical, Bloomington IN) that was explored (Lot #LB835143) is a multi-layer acellular ECM manufactured from animal derived non-cross linked collagen (porcine small intestinal submucosa; SIS). The second biologic wound matrix (MatriStem® Multilayer Wound Matrix, WSM0710; ACell Columbia MD) that was used is a multi-layer ECM manufactured from urinary bladder matrix (UBM) with an intact epithelial basement membrane and various collagens (porcine derived; Lot # MML4820-21). Prior to implantation each ECM was prepared following individual manufacture suggestions. Briefly, under sterile conditions the ECM was rehydrated in room temperature saline and trimmed to fit the VML defect site. The ECM was circumferentially anchored in place by ~12–15 interrupted and locking proline sutures. An alternative biomaterial negative control using Hya was explored. Hya is a naturally occurring component of the extracellular matrix; it is biodegradable, non-immunogenic and anti-adhesive. In this context the use of Hya as a hydrogel was to provide a barrier for cell migration within the VML defect site. A pre-polymer solution consisting of 1% weight/volume thiol-modified hyaluronan (Glycosil®, ESI BIO, Alameda, CA; Lot #15E028) and 1% PEGDA (MW 3.4kDA) in degassed sterile water was mixed, drawn into a 30 ml syringe, and subsequently allowed to cross-link at 37 °C for 30 minutes in a humidified incubator (5% CO_2_, 37 °C). Following the VML injury, the muscle fascia was sutured with interrupted and locking proline sutures and the Hya was placed in the fascia pocket with a 16 G syringe. For the sham operated and non-repaired limbs, sutures were placed to mark the muscle and VML defect, respectively (Supplementary Fig. 1). In the rat model, a 6 mm piece of Hya was placed into the surgically created VML defect and fascia was sutured with interrupted sutures.

#### In Vivo Functional Testing

The strength of the dorisflexor muscles of the anterior compartment in each leg of pigs was tested with the knee stabilized at 90° and activated by subdermal stimulation of the peroneal nerve using needle electrodes^47,48^. Maximal isometric tetanic torque was elicited using 100 Hz, 0.1 ms pulse, over 800 ms (Grass S88 stimulator and 890 A Aurora Scientific large animal strength testing apparatus). In all cases, torque was measured over a range of joint angles. Body weight was assessed immediately prior to each procedure and used to normalize torque values. When available the torque was also normalized by the muscle volume of the anterior compartment as determined by CT imaging.

To assess the function of the isolated rat TA muscle the distal tendons of the extensor digitorum longus and extensor hallicus longus muscles severed above the retinaculum^6^. The muscle was activated via subcutaneous needle electrodes placed on both sides of the common peroneal nerve and function was assessed with the ankle at a right angle using a dual-mode muscle lever system elicited using 0.1 ms pulse width over 400 ms across a range (10–200 Hz) of frequencies (Grass S88 and 305B Aurora Scientific servomotor).

#### Reliability of In Vivo Functional Testing

Prior to surgical procedure a subset of 8 pigs underwent functional testing to determine reliability and optimization of the *in vivo* testing parameters. In each pig, both the right and left limbs were tested for isometric torque across seven joint angles (neutral and 10°, 20°, 30°, 40°, 50°, and 55° of plantarflexion). At each angle tested, two or three measurements were taken, with the exception of 55° of plantarflexion when only one measurement was taken due to near end range of motion (plantarflexion 62.4 ± 1.3°). At each joint angle, isometric torque was normalized to body weight and evaluated to determine if differences existed between right and left limbs, the order of the limb tested, and minimum and maximum values obtained for all values. In the whole model (i.e., across all angles) there was no difference between right and left limbs (p = 0.972) or the order of limbs tested (p = 0.999). Furthermore, there were also no differences when examined at individual joint angles (Supplementary Fig. 2). Multiple data points at individual joint angles were used to determine coefficient of variation (CV) for each limb tested (Supplementary Fig. 2). Using a one way ANOVA any differences in the CV across joint angles was determined. There is greater variation at the neutral joint angle (0°) tested than at all other angles (Supplementary Fig. 2; p = 0.002), however this is likely due to optimization of electrode placement across the peroneal nerve at the beginning of the testing. Given the high measurement reliability, only one contraction per joint angle following optimization of electrode placement was tested on all retest days for this study, this reduction was ideal to reduce the subject exposure to anesthetic gas.

#### CT Imaging

Standard imaging studies of the lower limb were conducted using a Toshiba 160 slice CT at 0.5 mm slice thickness. Scans were taken prior to *in vivo* function testing 10 weeks post-VML. Additionally, a subset of animals (n = 6) underwent scans prior to surgical procedures and at 2 weeks post-surgery (n = 4). Scans were analyzed with Vitrea Vital Medical Imaging Software to estimate volume of the anterior compartment. The volume was estimated from the coronal 2D images and then reconstructed into a 3D model by an investigator blinded to experimental group. Volume measurements were normalized to body mass, additionally anterior compartment volume was used to normalize muscle torque.

#### Histological Analyses

Following the terminal *in vivo* functional assessment, samples from the PT, tibialis and digitorum synergist muscles were collected. In all cases muscle samples were excised using a 10 mm biopsy punch through the full thickness of the muscle and then frozen in melting isopentane and stored at −80 °C until analysis. Each muscle sample was cross-sectioned at 8 µm and stained with standard histologic procedures for hematoxylin and eosin and Masson’s trichrome. Bright filed images were acquired with 20x (plan-apochromat 0.80 N.A, working distance 0.55 mm) objective on a Zeiss Axio Scan.Z1 (Carl Zeiss Microscopy, Thornwood NY) and stitched into a large composite image. Composite images were saved separately as a 24-bit, 96 dpi color images. Additionally, an independent investigation of the hematoxylin and eosin and Masson’s trichrome staining of the skeletal muscle was conducted by a board certified pathologist blinded to experimental group. Narrative reports were provided for all muscle sections. Histologic samples acquired from the rat TA muscle in the pilot study were processed with the same approach. Quantitative assessments of Hya presence at 2 and 8 weeks post injury were performed by manually circling a region of interest over the observed material using MATLAB (Mathworks, Natick, MA).

Immunofluorescent staining of the PT muscle was conducted using a combination of sarcomeric MyHC (MF20 obtained from the Developmental Studies Hybridoma Bank, mouse monoclonal IgG2b; 5 µg/ml), laminin (Abcam Ab11576, rat monoclonal IgG1; 2.5 µg/ml), Pax7 (Pax7 obtained from the Developmental Studies Hybridoma Bank, mouse monoclonal IgG1κ; 5 µg/ml), CD163 (Novus Biologicals NB110-40686, mouse monoclonal IgG1, 5 µg/ml) DAPI (Molecular Probes D21490, 1 µg/ml), and Wheat Germ Agglutinin (Molecular Probes W32466 647-conjugated, 1 µg/ml). Appropriately paired secondary antibodies at a dilution of 1:200 (Invitrogen A21121, A21141, or A11007) were subsequently used. In all cases the expected staining patterns in normal skeletal muscle were observed and the specificity of anti-labeling was confirmed by the absence of staining outside expected structures and was consistent with manufacturer’s technical information.

Confocal imaging was conducted with an Olympus FluoView FV1000 laser scanning confocal microscope (Olympus America Inc., Melville, NY) mounted on an inverted Olympus IX81 microscope and equipped with Multi-line Argon (458, 488, and 515 nm), HeNeG (543 nm), diode (405 nm) and diode (635 nm) lasers using an Olympus UPLSAPO 20x/0.85 N.A. or 60x/1.35 N.A. oil immersion lenses. Images were acquired in a 512 × 512 array with pixel dimensions (0.5 × 0.5 μm). Confocal images were saved as individual channel 12-bit multi-TIFF files, and reconstructed in MetaMorph (Molecular Devices LLC., Sunnyvale CA). For display purposes only images were down-converted, without introducing any changes in brightness or contrast. Laser intensity was held consistent across imaging of the same antibody probes. During all imaging and investigation, investigators were blinded to experimental group.

#### Rat RNA

Real time reverse transcription quantitative polymerase chain reaction (RT-qPCR) was used to quantify gene expression within the VML defect region at 2 weeks post-injury. RNA was isolated from the homogenized tissue using the TRIzol method. The RNA samples were reverse transcribed into cDNA using a QuantiTech Rev Transcription kit (Qiagen, Hilden, Germany) according to the manufacturer’s protocol. Gene expression for target markers was determined using custom-designed primers (Supplementary Fig. 3) with RT-qPCR amplification performed in the presence of SYBR Green (Bio-Rad Laboratories, Hercules, CA). The raw fluorescence data was processed using LinRegPCR (v12.11; http://www.hartfaalcentrum.nl) with glyceraldehyde-3-phosphate dehydrogenase (GAPDH) serving as the endogenous control^49^.

#### RNA Sequencing

During the harvest for histologic samples, samples from the PT muscle were collected for whole tissue RNA isolation. The muscles were thawed, minced and homogenized (Tissue Ruptor, Qiagen) for 30 seconds at room temperature and total RNA was isolated from the slurry using the miRNeasy Mini Kit (Qiagen) as per the manufacturer’s instructions. RNA concentration and integrity were measured with a Nanodrop spectrophotometer (Nanodrop 2000c) and Bioanalyzer (Agilent 2100), respectively. 1 μg of isolated total RNA was used to produce cDNA libraries using the Truseq protocol (Illumina), as per the manufacturer’s instructions. Biological replicates or triplicates were produced from each condition and individual libraries were pooled and sequenced using 76 base-pair (bp) paired-end reads on a Hi-Seq. 2500 in high output mode to an average depth of 55 M reads per library.

#### Data Processing

High-throughput sequencing reads were pseudoaligned to the University of California, Santa Cruz, annotated *Sus scrofa* (Pig) genome (build 9.2) and transcript abundances were quantified using Kallisto^50^ with default parameters. Differential expression (DE) of transcripts was calculated using the R/Bioconductor implementation of DESeq. 2^51^. Analysis of expression data, such as set analysis based on treatment specific expression profiles, principal component analysis, and correlation of inter-treatment gene expression were performed with R. Count data was log2 transformed, after adding a pseudocount of 0.5 to all measurements. Differential gene expression (relative to sham controls) in gene matrixes and plots were called significant at a False Discovery Rate (FDR; after Benjamini-Hochberg correction) of less than 0.05. Given the high number of genes exhibiting significant differential expression, additional filters were set to obtain DE genes for further analysis (1 transcript per kilobase million in all of the treatment group samples and have a log2 fold change of at least 1 in at least one treatment group). Treatment-group-specific DE genes were computed and set analysis was used to determine overlapping and unique genes between groups using base R libraries and plotted using R package VennDiagram (version 1.6.17). Principal component analysis was performed on significant DE genes using base R libraries and plotted using ggplot2 (version 2.1.0).

Significant DE genes were also used to compute Gene Set Enrichment Analyses. Gene Sets used for enrichment analyses were obtained from the Gene Ontology Consortium^52^, REACTOME^53^, and BioCyc^54^. GO terms were limited to Biological Processes. Pathway analysis was conducted on qualified genes using the R/Bioconductor package GAGE^55^ and significant (q-value < 0.05) pathways were ranked in order of significance. Pathways selected were within the top 50 ranked up- or down-regulated pathways and reflected general trends with those top 50 pathways. A matrix of treatment group x treatment group Pearson correlation values, generated using treatment expression profiles and base R libraries, was calculated and plotted using the R package pheatmap (version 1.0.8).

### Data availability

RNA-Seq datasets are uploaded onto GEO and accession numbers are pending.

### Statistical analysis

All quantitative data was analyzed using JMP (version 10.0 SAS Institute, Inc., North Carolina). Treatment group sample sizes of at least 3 were determined prior to the study to allow for the detection of a 33% increase in muscle torque production at power (1 – ß) of 0.80 and α of 0.05, with a shared standard deviation of 0.025 Nm/kg determined in a functional optimization study. Body weight was analyzed by one-way repeated measures ANOVA (time; repeated) over the 12 weeks study. Pre-surgical data was analyzed one-way ANOVA to determine any differences existed across groups, no differences in pre-surgical data were determined (p ≥ 0.070) and therefore pre-surgical data was collapsed across experimental groups. Data was analyzed by one-way ANOVA (group) for anterior compartment volume, torque, RT-PCR, and Hya degradation, or two-way ANOVA for peak torque over time or stimulation frequency (group x time point/frequency). Tukey’s post-hoc analyses were performed upon significant ANOVA. Statistical handling of RNAseq data are described above. Data are reported as mean ± SE and significance was determined at the α < 0.05 level.

### Disclosures

The opinions or assertions contained here are the private views of the authors and are not to be construed as official or as reflecting the views of the Department of the Army, the Department of Defense, nor the U.S. Government.

## Electronic supplementary material

Supplementary Data

## References

[1] Garg K (2015). Volumetric muscle loss: persistent functional deficits beyond frank loss of tissue. Journal of orthopaedic research: official publication of the Orthopaedic Research Society.

[2] Mase VJ (2010). Clinical application of an acellular biologic scaffold for surgical repair of a large, traumatic quadriceps femoris muscle defect. Orthopedics.

[3] Corona BT, Rivera JC, Owens JG, Wenke JC, Rathbone CR (2015). Volumetric muscle loss leads to permanent disability following extremity trauma. J Rehabil Res Dev.

[4] Rivera, J. C. & Corona, B. T. Muscle-related Disability Following Combat Injury Increases With Time. *U.S. Army Medical Department journal*, 30–34 (2016). 26874094

[5] Ward CL (2016). Autologous Minced Muscle Grafts Improve Muscle Strength in a Porcine Model of Volumetric Muscle Loss Injury. Journal of orthopaedic trauma.

[6] Corona BT (2013). Autologous minced muscle grafts: a tissue engineering therapy for the volumetric loss of skeletal muscle. Am J Physiol Cell Physiol.

[7] Kasukonis B (2016). Codelivery of Infusion Decellularized Skeletal Muscle with Minced Muscle Autografts Improved Recovery from Volumetric Muscle Loss Injury in a Rat Model. Tissue Eng Part A.

[8] Wolf MT, Dearth CL, Sonnenberg SB, Loboa EG, Badylak SF (2015). Naturally derived and synthetic scaffolds for skeletal muscle reconstruction. Advanced drug delivery reviews.

[9] Badylak SF, Dziki JL, Sicari BM, Ambrosio F, Boninger ML (2016). Mechanisms by which acellular biologic scaffolds promote functional skeletal muscle restoration. Biomaterials.

[10] Corona BT, Greising SM (2016). Challenges to acellular biological scaffold mediated skeletal muscle tissue regeneration. Biomaterials.

[11] Turner NJ (2010). Xenogeneic extracellular matrix as an inductive scaffold for regeneration of a functioning musculotendinous junction. Tissue Eng Part A.

[12] Dziki, J. *et al*. An acellular biologic scaffold treatment for volumetric muscle loss: results of a 13-patient cohort study. *Npj Regenerative Medicine* **1**, 16008, 10.1038/npjregenmed.2016.8http://www.nature.com/articles/npjregenmed20168 - supplementary-information (2016). 10.1038/npjregenmed.2016.8 PMC5744714 29302336

[13] Klein CS, Marsh GD, Petrella RJ, Rice CL (2003). Muscle fiber number in the biceps brachii muscle of young and old men. Muscle Nerve.

[14] MacDougall JD, Sale DG, Alway SE, Sutton JR (1984). Muscle fiber number in biceps brachii in bodybuilders and control subjects. Journal of applied physiology: respiratory, environmental and exercise physiology.

[15] Sicari BM (2014). An acellular biologic scaffold promotes skeletal muscle formation in mice and humans with volumetric muscle loss. Science translational medicine.

[16] Grogan, B. F. & Hsu, J. R. Volumetric muscle loss. *J Am Acad Orthop Sur*g **19** Suppl 1, S35-37, doi:19/suppl_1/S35 [pii] (2011). 10.5435/00124635-201102001-00007 21304045

[17] Corona, B. T., Wenke, J. C. & Ward, C. L. Pathophysiology of volumetric muscle loss injury. *Cells Tissues Organs*, 10.1159/000443925. 10.1159/000443925 27825160

[18] Corona BT (2013). The promotion of a functional fibrosis in skeletal muscle with volumetric muscle loss injury following the transplantation of muscle-ECM. Biomaterials.

[19] Aurora A, Roe JL, Corona BT, Walters TJ (2015). An acellular biologic scaffold does not regenerate appreciable de novo muscle tissue in rat models of volumetric muscle loss injury. Biomaterials.

[20] Shu XZ (2004). Attachment and spreading of fibroblasts on an RGD peptide-modified injectable hyaluronan hydrogel. Journal of biomedical materials research. Part A.

[21] Gentile NE (2014). Targeted rehabilitation after extracellular matrix scaffold transplantation for the treatment of volumetric muscle loss. American journal of physical medicine & rehabilitation/Association of Academic Physiatrists.

[22] Han N (2016). Electrodiagnostic Evaluation of Individuals Implanted With Extracellular Matrix for the Treatment of Volumetric Muscle Injury: Case Series. Physical therapy.

[23] Serrano AL (2011). Cellular and molecular mechanisms regulating fibrosis in skeletal muscle repair and disease. Current topics in developmental biology.

[24] Murphy MM, Lawson JA, Mathew SJ, Hutcheson DA, Kardon G (2011). Satellite cells, connective tissue fibroblasts and their interactions are crucial for muscle regeneration. Development.

[25] Lemos DR (2015). Nilotinib reduces muscle fibrosis in chronic muscle injury by promoting TNF-mediated apoptosis of fibro/adipogenic progenitors. Nature medicine.

[26] Brack AS (2007). Increased Wnt signaling during aging alters muscle stem cell fate and increases fibrosis. Science (New York, N.Y.).

[27] Sadtler K (2016). Developing a pro-regenerative biomaterial scaffold microenvironment requires T helper 2 cells. Science (New York, N.Y.).

[28] Lepper, C., Partridge, T. A. & Fan, C. M. An absolute requirement for Pax7-positive satellite cells in acute injury-induced skeletal muscle regeneration. *Development* **138**, 3639-3646, 138/17/3639 [pii]10.1242/dev.067595 (2011). 10.1242/dev.067595 PMC3152922 21828092

[29] Sambasivan R (2011). Pax7-expressing satellite cells are indispensable for adult skeletal muscle regeneration. Development.

[30] Ma J, Baker AR, Calabro A, Derwin KA (2017). Exploratory study on the effect of osteoactivin on muscle regeneration in a rat volumetric muscle loss model. PLoS One.

[31] Corona BT, Ward CL, Baker HB, Walters TJ, Christ GJ (2014). Implantation of *in vitro* tissue engineered muscle repair constructs and bladder acellular matrices partially restore *in vivo* skeletal muscle function in a rat model of volumetric muscle loss injury. Tissue Eng Part A.

[32] Chen XK, Walters TJ (2013). Muscle-derived decellularised extracellular matrix improves functional recovery in a rat latissimus dorsi muscle defect model. Journal of plastic, reconstructive & aesthetic surgery: JPRAS.

[33] Garg K, Ward CL, Rathbone CR, Corona BT (2014). Transplantation of devitalized muscle scaffolds is insufficient for appreciable de novo muscle fiber regeneration after volumetric muscle loss injury. Cell and tissue research.

[34] Aurora A, Garg K, Corona BT, Walters TJ (2014). Physical rehabilitation improves muscle function following volumetric muscle loss injury. BMC sports science, medicine and rehabilitation.

[35] Garg K, Corona BT, Walters TJ (2014). Losartan administration reduces fibrosis but hinders functional recovery after volumetric muscle loss injury. Journal of applied physiology (Bethesda, Md.: 1985.

[36] Arnold L (2007). Inflammatory monocytes recruited after skeletal muscle injury switch into antiinflammatory macrophages to support myogenesis. The Journal of experimental medicine.

[37] Aguilar CA (2015). *In vivo* Monitoring of Transcriptional Dynamics After Lower-Limb Muscle Injury Enables Quantitative Classification of Healing. Scientific reports.

[38] Aguilar CA (2016). Transcriptional and Chromatin Dynamics of Muscle Regeneration after SevereTrauma. Stem cell reports.

[39] Naito AT (2012). Complement C1q activates canonical Wnt signaling and promotes aging-related phenotypes. Cell.

[40] Mann CJ (2011). Aberrant repair and fibrosis development in skeletal muscle. Skeletal muscle.

[41] Desguerre I (2012). A new model of experimental fibrosis in hindlimb skeletal muscle of adult mdx mouse mimicking muscular dystrophy. Muscle Nerve.

[42] Ma J, Sahoo S, Baker AR, Derwin KA (2015). Investigating muscle regeneration with a dermis/small intestinal submucosa scaffold in a rat full-thickness abdominal wall defect model. Journal of biomedical materials research. Part B, Applied biomaterials.

[43] Turner NJ, Badylak JS, Weber DJ, Badylak SF (2012). Biologic scaffold remodeling in a dog model of complex musculoskeletal injury. J Surg Res.

[44] Grasman JM, Do DM, Page RL, Pins GD (2015). Rapid release of growth factors regenerates force output in volumetric muscle loss injuries. Biomaterials.

[45] VanDusen KW, Syverud BC, Williams ML, Lee JD, Larkin LM (2014). Engineered skeletal muscle units for repair of volumetric muscle loss in the tibialis anterior muscle of a rat. Tissue Eng Part A.

[46] Rossi, C. A. *et al*. *In vivo* tissue engineering of functional skeletal muscle by freshly isolated satellite cells embedded in a photopolymerizable hydrogel. *FASEB J* **25**, 2296-2304, fj.10-174755 [pii] 10.1096/fj.10-174755 (2011). 10.1096/fj.10-174755 21450908

[47] Kheirabadi BS (2014). Long-term effects of Combat Ready Clamp application to control junctional hemorrhage in swine. The journal of trauma and acute care surgery.

[48] Ward, C. L. *et al*. Autologous minced muscle grafts improve muscle strength in a pourcine model of volumetric muscle loss injury. *J Orthop Trauma* In Press (2016). 10.1097/BOT.0000000000000673 27466826

[49] Ruijter JM (2009). Amplification efficiency: linking baseline and bias in the analysis of quantitative PCR data. Nucleic acids research.

[50] Bray NL, Pimentel H, Melsted P, Pachter L (2016). Near-optimal probabilistic RNA-seq quantification. Nat Biotechnol.

[51] Love MI, Huber W, Anders S (2014). Moderated estimation of fold change and dispersion for RNA-seq data with DESeq. 2. Genome biology.

[52] Ashburner M (2000). Gene ontology: tool for the unification of biology. The Gene Ontology Consortium. Nature genetics.

[53] Fabregat A (2016). The Reactome pathway Knowledgebase. Nucleic acids research.

[54] Caspi R (2014). The MetaCyc database of metabolic pathways and enzymes and the BioCyc collection of Pathway/Genome Databases. Nucleic acids research.

[55] Luo W, Friedman MS, Shedden K, Hankenson KD, Woolf PJ (2009). GAGE: generally applicable gene set enrichment for pathway analysis. BMC Bioinformatics.

