# Supplementary material for: Unwavering Pathobiology of Volumetric Muscle Loss Injury
Source: Sci Rep. 2017 Oct 13;7:13179. doi: 10.1038/s41598-017-13306-2 (PMC5640632; doi:10.1038/s41598-017-13306-2)
Supplement: Supplementary file 1 — Supplementary Data [file 41598_2017_13306_MOESM1_ESM.pdf]

Unwavering Pathobiology of Volumetric Muscle Loss Injury

Sarah M Greising[1], Jessica C Rivera[1], Stephen M Goldman[1], Alain Watts[2], Carlos A Aguilar[2,3], & Benjamin T Corona[1]*

[1]Extremity Trauma and Regenerative Medicine, United States Army Institute of Surgical Research, Fort Sam Houston, TX; [2]Massachusetts Institute of Technology, Lincoln Laboratory, Lexington, MA; Departments of [3]Biomedical Engineering, University of Michigan, Ann Arbor, MI

Supplementary Information

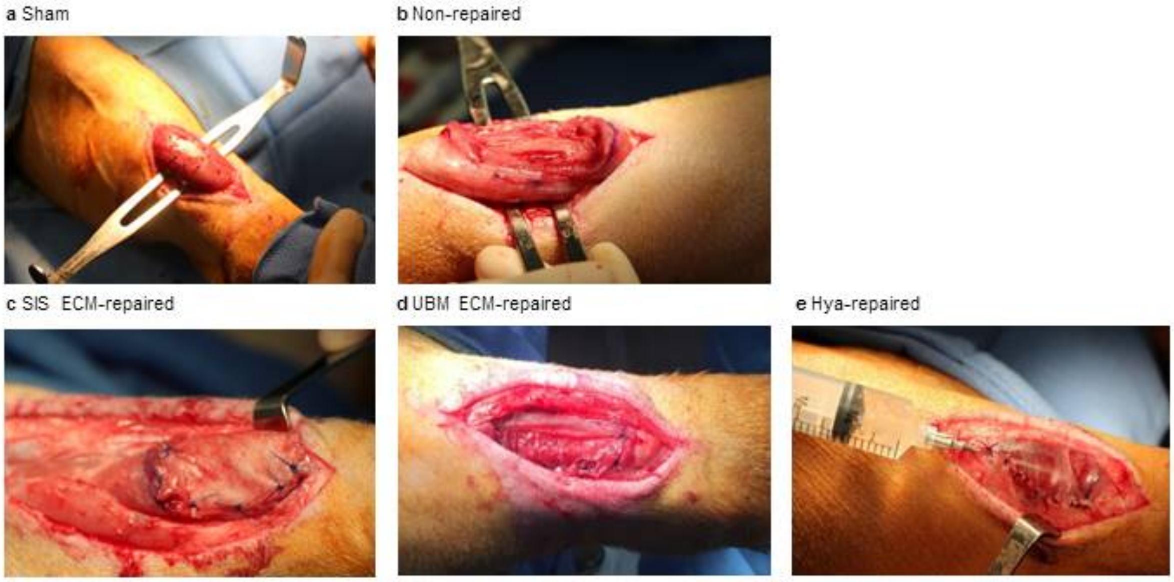

Supplemental Figure 1 A VML injury was surgically created bilaterally in the porcine peroneous tertius (PT) muscle, the primary dorsiflexor muscle. Intraoperative picture of the VML procedure demonstrate the removal of ~5 g of muscle representing a ~3x3x1 cm area from the middle third of the PT muscle. Limbs were randomized to (**a**) sham VML procedures with the muscle remaining (**b**) non-repaired, or (**c-e**) the implantation of a biomaterial into the VML defect. Randomization also occurred for the biomaterial implanted, (**c**) SIS, (**d**) UBM, or (**e**) Hya.

Supplemental Figure 2 Reliability of *in vivo* strength assessments. Muscle function was determined by peroneal nerve stimulation *in vivo*, anterior muscle compartment torque was analyzed prior to surgery for optimization and validation in a subset of animals (n=8).

Optimization of *in vivo* functional testing

Joint Angle	Right vs. Left<br>p-value	Limb order tested<br>p-value	Maximum vs. minimum value<br>p-value
0° (neutral)	0.942	0.999	0.202
10°	0.469	0.999	0.148
20°	0.198	0.999	0.632
30°	0.868	0.999	0.498
40°	0.943	0.999	0.584
50°	0.430	0.999	0.535

Data analyzed by one-way ANOVA

Variation of *in vivo* functional testing

Joint Angle	Mean Normalized Torque ± SE<br>(Nm/kg)	CV mean ± SE<br>(%)
0° (neutral)	0.119 ± 0.006	12.4 ± 4.0 *
10°	0.187 ± 0.009	8.6 ± 2.0
20°	0.244 ± 0.009	3.6 ± 1.4
30°	0.274 ± 0.009	2.9 ± 0.7
40°	0.272 ± 0.008	2.6 ± 0.7
50°	0.245 ± 0.008	2.5 ± 1.2
55°	0.223 ± 0.010	--

Torque is normalized to body weight; Data analyzed by one-way ANOVA (p=0.002; *significantly different than all other joint angles)

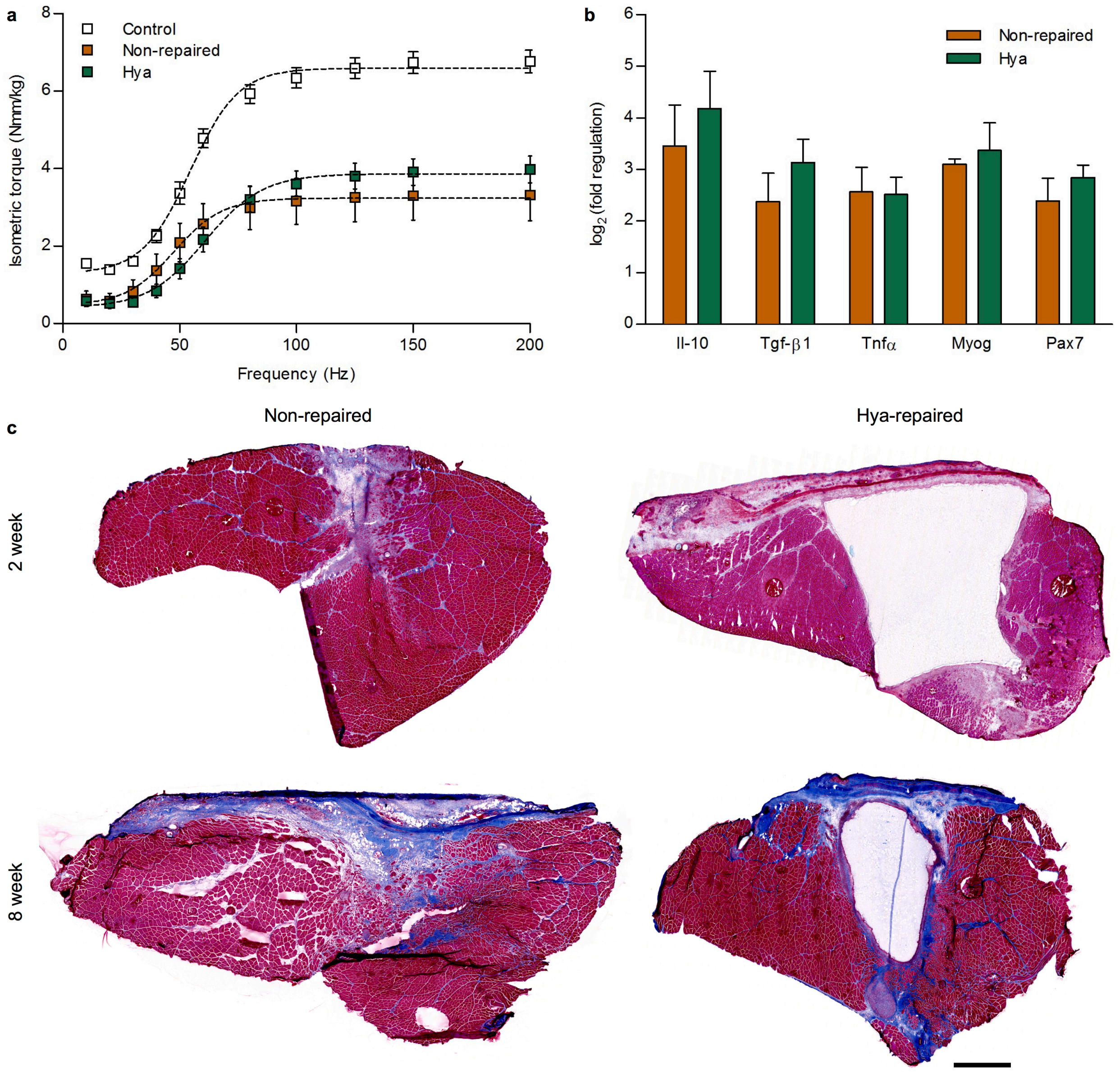

d

	Forward sequence	Reverse sequence	Amplicon length
IL-10	5′-CCCAGAAATCAAGGAGCATT-3′	5′-GCTCCACTGCCTTGCTTTTA-3′	116
Tgf-β1	5′-GTCAGACATTCGGGAAGCA-3′	5′-CCAAGGTAACGCCAGGAAT-3′	138
Tnfα	5′-ACTCGAGTGACAAGCCCGTA-3′	5′-CCTTGTCCCTTGAAGAGAACC-3′	184
Myog	5′-CTACAGGCCTTGCTCAGCTC-3′	5′-GTTGGGACCAAACTCCAGTG-3′	153
Pax7	5′-GCAGTCGGACCACATTCAC-3′	5′-CGCACGACGGTTACTGAAC-3′	155

Supplemental Figure 3 Hya repair does not orchestrate functional skeletal muscle tissue regeneration in a rat TA VML injury model supporting use of Hya as a negative biomaterial control for analysis in the porcine PT VML injury. (**a**) *In vivo* torque-frequency relationship of the isolated TA muscle for contralateral control (n=7), non-repaired (n=3), and Hya-repaired (n=7) at 8 weeks post-injury. There is a persistent strength deficit in the non-repaired muscles,

that is not significantly different than that of the Hya-repaired muscles across stimulation frequencies ($p \geq 0.165$). (**b**) Gene expression of inflammatory cytokine and myogenic transcription factors was analyzed at 2 weeks post-injury in the injured TA muscle relative to the contralateral limb ($n=3$ per transcript). There was similar upregulation of genes in the non- and Hya-repaired TA muscles ($p \geq 0.421$). (**c**) Representative histologic evaluation of Masson's trichrome muscle sections do not display any evidence of muscle tissue regeneration in the defect area of the TA muscle. Of note there is expected degradation of the Hya from 2 to 8 weeks post-injury. Scale bar is 1 mm. (**d**) Primer sequences used for transcript analysis.

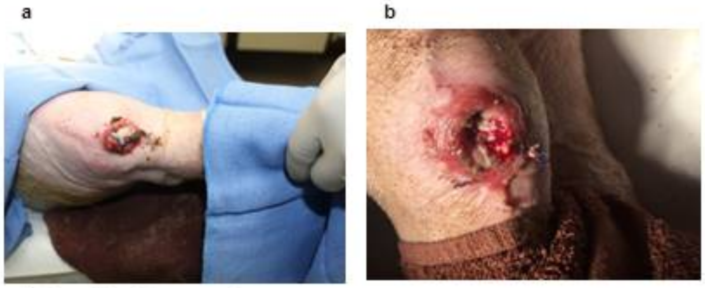

c Noteworthy adverse events

	Sham n=2	Non-repaired n=12	SIS n=5	UBM n=4	Hya n=3
Prolonged inflammation	0	0	5	0	0
Wound dehiscence	0	1	3	0	0
Erosion	0	0	1	0	0
Seroma	0	0	4	0	0
Additional pain and/or anti-inflammatory medication needed	0	0	4	0	0
Loss of biomaterial components and/or ECM explant necessary	NA	NA	3	0	0

Supplemental Figure 4 SIS repair presented wound healing complications. (**a**) At the 2 week function check the bandage was removed and the ECM was completely exposed where the implanted material eroded through the skin lateral to the surgical incision. The wound was debrided, irrigated, and the limb was re-bandaged. (**b**) Three days later the bandage was removed to again address the wound. At this point the wound was still open and there were noted signs of inflammation. Again the wound was debrided, irrigated, at this point the remaining SIS was completely removed, and the limb was re-bandaged. (**c**) All noteworthy adverse events that occurred in the present study are noted; n's listed represent totals included for the study.

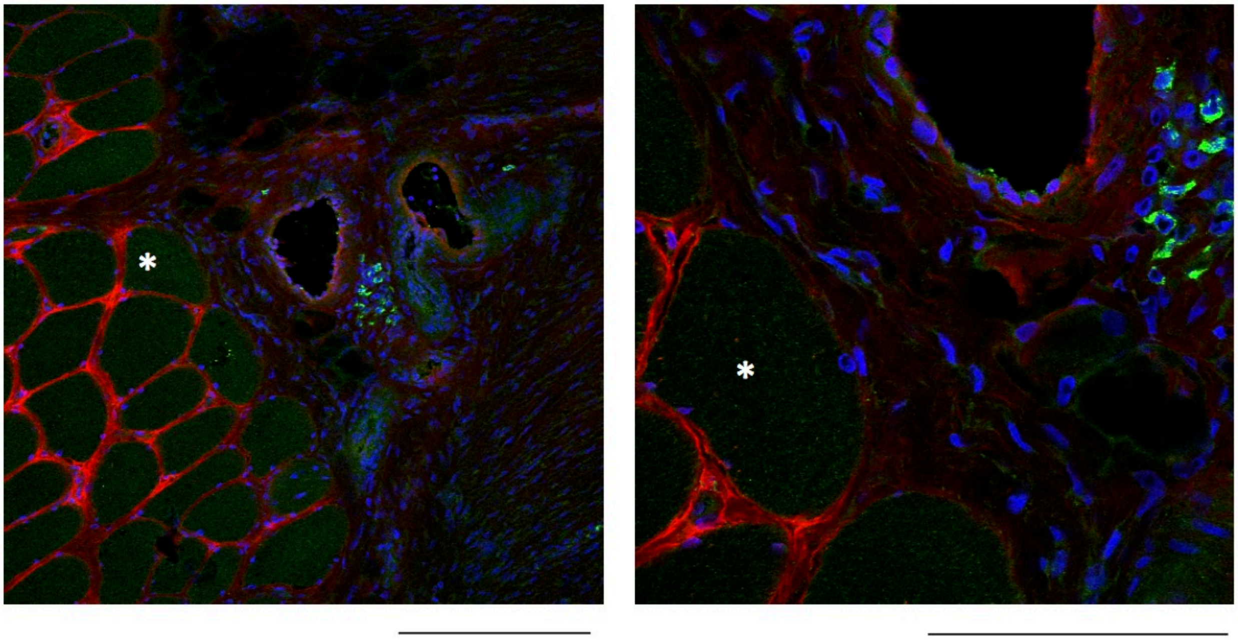

Supplemental Figure 5 Pax7 positive cell clusters in close proximity to remaining muscle mass. Pax7 positive cells were observed in two muscles repaired with SIS. The clusters were in close proximity the remaining muscle mass and not observed further in the defect area. The left (scale bar = 200 µm) and right (scale bar = 100 µm) panel are derived from the same muscle at the interface of the remaining muscle mass and the defect area. * demarks the same muscle fiber in each panel. Pax7, green; WGA, red; DAPI, blue.

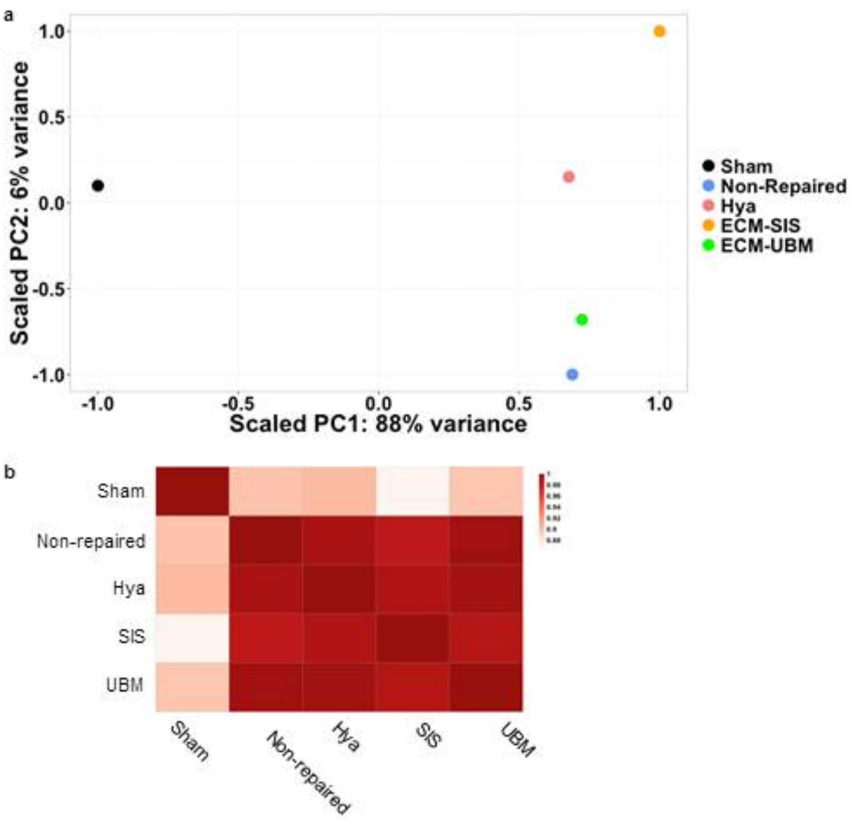

Supplemental Figure 6 Transcriptional analysis indicated that healthy muscle (sham operated) was extensively dissimilar than those of VML injured or repaired muscles. (**a**) Principal component analysis (PCA) of transcriptome analyses shows significant separation of the sham tissues with those that were VML injured, regardless of repair. (**b**) Pearson correlation coefficient matrix of tissue transcriptomes for both uninjured and VML injured regardless of treatment.
